# Passive Exoskeleton with Gait-Based Knee Joint Support for Individuals with Cerebral Palsy

**DOI:** 10.3390/s22228935

**Published:** 2022-11-18

**Authors:** Maxwell Kennard, Hideki Kadone, Yukiyo Shimizu, Kenji Suzuki

**Affiliations:** 1 School of Integrative and Global Majors, University of Tsukuba, Tsukuba 305-8577, Japan; 2 Center for Cybernics Research and Center for Innovative Medicine and Engineering, University of Tsukuba Hospital, Tsukuba 305-8577, Japan; 3 Department of Rehabilitation Medicine, Faculty of Medicine, University of Tsukuba, Tsukuba 305-8577, Japan; 4 Faculty of Systems, Information and Engineering, University of Tsukuba, Tsukuba 305-8577, Japan

**Keywords:** wearable robotics, exoskeletons, assistive devices

## Abstract

Cerebral palsy is a neurological disorder with a variety of symptoms that can affect muscle coordination and movement. Crouch gait is one such symptom that is defined as excessive knee flexion accompanied by a crouched posture. This paper introduces a passive exoskeleton to support the knee joint during stance of individuals with cerebral palsy that are affected by crouch gait. The exoskeleton utilizes a hydraulic disc brake mechanism that is actuated only by the body weight and gait of the wearer to provide a braking torque at the knee joint. This passive, gait-based control method aims to offer a compact, lightweight, and simple alternative to existing exoskeletons. Preliminary experiments were conducted to verify the mechanics, safety, and braking capabilities of the device with healthy participants. A pilot study with an individual with cerebral palsy was then conducted. The individual with cerebral palsy showed a reduction in hip joint angle when using the device (18.8${}^{\circ }$ and 21.7${}^{\circ }$ for left and right sides, respectively). The muscle co-activation index was also reduced from 0.48 to 0.24 on the right side and from 0.17 to 0.017 on the left side. However, changes such as activation timing and device training need to be improved to better support the user.

## 1. Introduction

Cerebral palsy (CP) refers to a group of neurological disorders that permanently affect body movement and muscle coordination [1]. As the most common motor disability found in children, the number of diagnoses of this condition is around 2 to 3 per 1000 children in the US [2]. Crouch gait, muscle spasticity, and lack of coordination are some of the symptoms experienced due to CP [3]. Unfortunately, without a cure, the best that can be currently undertaken for these children is to try to improve their quality of life with treatments and therapy. Even with these options, there is still no consistent and reliable method for aiding those with CP [4]. Moreover, if these methods do prove beneficial for a particular patient, there is no guarantee that their condition will not recess after the completion of the treatment [5]. It is estimated that during the life of an individual with CP, the costs of therapy and treatments can total an average of USD 921,000 [6]. There is a clear need for economically viable and effective treatment options.

Crouch gait is one of the more common conditions experienced by individuals with CP. This gait impairment is identified from the crouched posture and excessive hip and knee flexion during stance [7]. The stance phase is roughly 60% of the gait cycle and defined as the moment the foot contacts the ground, heel strike, to the moment the foot leaves the ground, toe-off [8]. During stance the leg must be able to support the full weight of the body. This becomes difficult for individuals with crouch gait because of excessive knee flexion. A healthy knee typically flexes about 15${}^{\circ }$ during stance, but an individual with crouch gait may experience flexion ranging from 20${}^{\circ }$ to 40${}^{\circ }$ [9]. Crouch gait is also a less efficient gait and requires more energy to move than a healthy gait [10]. Due to the various aspects of crouch gait, individuals with CP require extra support during stance to aide them in walking.

This paper proposes a passive exoskeleton that can provide a braking force to the knee joint during stance that is actuated by the user’s body weight and gait. This work contributes to the field of wearable exoskeletons in three points: (i) verifying the performance of adding a braking force to the knee joint only during stance; (ii) proposing a compact, lightweight, and simple mechanism for supporting exoskeleton joints; and (iii) reporting on the feasibility and effects of a gait-based control system for an individual with CP. This paper is a continuation of our work that was presented in a non-reviewed abstract [11]. The new contributions are the system modeling, brake torque and activation timing experiments, and pilot study.

## 2. Related Work

Recently, exoskeleton robots have been shown to improve crouch gait in children with CP. Lerner et al. developed an active exoskeleton that was able to show significant improvements in both knee extension and range of motion [12]. The device was tested on a 6-year-old boy and weighed 3.2 kg. Additionally, the exoskeleton could only operate with a tethered power supply or a 0.75 kg battery for one hour. Yamada et al. created an active exoskeleton targeting CP for children [13]. This exoskeleton showed potential for assisting CP, but the sensing system needed improvement for more accurate gait detection. The device, weighing 1.76 kg, utilized three separate batteries to power an on-board microcontroller and motor. DC motors are not the only way to aid the gait pattern of a patient with CP. Shideler et al. took a hybrid approach when develop their exoskeleton. The exoskeleton did have motors, but they were only used to overcome the friction and inertia of the mechanism. The real support came from the use of Neuromuscular Electrical Stimulation (NMES) to stimulate the leg muscles during certain portions of the gait cycle [14]. This stimulation method showed promising results and they reported immediate results in the improvement of the knee flexion during the stance phase. The control methodology requires an initial calibration while tethered to a computer along with the use of a finite state machine to detect gait phase. This leaves the possibility for multiple points of failure and the participant needed to hold an emergency stop mechanism while performing the tasks for their safety.

Commercial products have also been developed to aid those with gait impairments. The C-Brace (Ottobock) uses a microprocessor to detect the knee angle and control the resistance provided by a hydraulic knee joint mechanism [15]. However, the C-Brace costs an average of USD 75,000 and requires professional fitting and training sessions before use. The Hybrid Assistive Limb (HAL) is another commercial device that has been studied and marketed for rehabilitation strategies involving stroke, spinal cord injury, and cerebral palsy [16]. The HAL exoskeleton detects the bio-electric muscle signals of the wearer and is able to anticipate and provide support for movement. In combination with a surgical procedure prior to use, the HAL was shown enhance the walking ability of a person with CP. Though the device is officially commercially available, it does not rent the device to individual users. Instead prospective patients are forced to find a hospital or associated partner for a chance at actually using the device. Roadblocks such as this one are another issue that individuals with CP may face when trying to find treatment options.

Active robot exoskeletons can typically offer more support, but passive devices are commonly more robust, lightweight, and economically viable. Several passive exoskeletons have been developed for lower extremity support. These devices aim to utilize a gait-based control system to relieve compressive loads on the knee joint. Wang et al. uses a compliant knee joint mechanism coupled with a support spring located at the heel of the user’s foot [17]. They were able to show a comparable reduction in knee joint forces between their passive mechanism and other active exoskeleton devices. Van Dijk et al. created an artificial tendon that connects the hip, knee, and ankle to minimize the work required by the joints during gait [18]. Unlike the theoretical calculations, the experimental results of this study did not show any significant reduction in energy usage. Due to lacking any active mechanisms, passive exoskeletons need both an energy storage component and a passive gait sensing component to maximize the support that can be provided. Even with these two components, there are mechanical limitations to the provided support when adjusting for different users. To adapt to a different user’s weight, a passive exoskeleton may need to redesign or replace the actuator entirely. An active exoskeleton can overcome this limitation with motors or other active components. There is also the issue of control. Passive devices typically utilize an ‘always-on’ control scheme. That is to say that the support is always provided when the device is being worn. This is usually the intended design of the mechanism, but may limit the user from performing certain actions. An active exoskeleton can easily adjust or disable the support to allow the user to perform actions outside of the normal intended sequence. For example, the user could disable the control system to allow them to bend down and pick up an object. This may not be possible with a passive exoskeleton unless the mechanism is specifically designed for it. In general, passive exoskeletons are less adjustable than active exoskeletons for accommodating different users and scenarios. The previously mentioned exoskeletons have been summarized in Table 1.

## 3. Materials and Methods

### 3.1. Concept

The main concept is a support mechanism that will automatically engage during the stance phase to apply a braking force to the knee joint. It then disengages during the swing phase. Applying a braking force to the knee joint could support the user’s body weight and reduce the burden on the muscles. Therefore, if proper support can be provided to the knee joint, the device could allow for improved gait among CP patients.

It could be argued that the sensing is the most important aspect of a robotic support exoskeleton. There are many reliable and unique mechanisms for physically supporting a human, but if these mechanisms are not actuated at the correct time, they could end being hindering or even harming the user. The previously mentioned active exoskeletons all relied on complex sensors, control loops, and models to determine when to best provide support to the user, while these can be great methods, computational errors or power failures are not out of the ordinary. Many active exoskeletons provide only discrete force. The support is either turned on or off depending on the gait phase of the user. Passive sensing allows for more continuous and proportional resistance to be applied to the desired joint.

Additionally, an often overlooked aspect of active control is how it makes the user feel. Kirkwood et al. expands on this idea in their research [19]. They state that “synchronization between exoskeleton suits and wearers is one of the most challenging requirements to operate these technologies effectively”. They go on to explain that events such as an active exoskeleton lagging behind the desired movements of the user or making unintended motions can make someone feel as if they are not fully in control. This can be very disturbing for a user when operating an exoskeleton. Instead we would like the user to feel more natural and in control when wearing our device. Therefore, in this study we propose a gait-based, passive sensing solution to control the support mechanism of the exoskeleton. It is our assumption that passive sensing can lead to better synchronization and give the user more confidence in the device, while still providing sufficient physical support.

**Figure 1:**
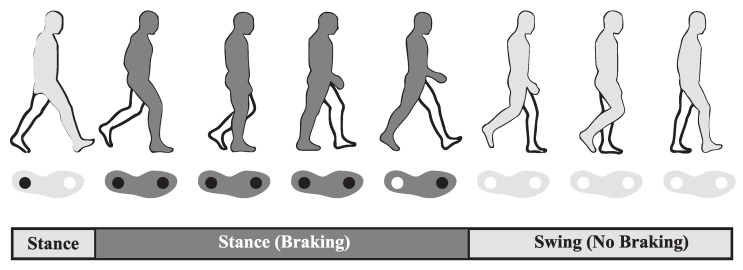
Brake actuation timing with respect to the gait cycle. The light gray color is the period during gait where the brake is not active and the leg is able to swing freely. The dark gray color is the period where the brake is active. The ○ and ● symbols show the locations on the foot of no applied pressure and applied pressure during the corresponding gait phase.

The exoskeleton was designed such that the activation of the support mechanism was entirely gait operated without any external sensing controllers. The gait-based control system has a caliper and rotor located collinear to the axis of rotation of the knee joint. A brake actuator is located on the sole of the foot near the metatarsals. Upon contact with the ground, the actuator’s pushrod depresses under the body weight of the user and pressurizes a hydraulic line connected to the caliper. This pressure provides the actuation that engages the piston inside of the caliper and locks the rotor in place. The braking of the rotor provides a braking force to the knee during stance. As the leg is lifted, the pushrod is able to extend, release the rotor, and allow the knee joint to bend freely again. Therefore, the knee joint braking mechanism automatically engages during the stance phase and dis-engages during the swing phase, assuming proper contact of the pushrod with the ground. The theoretical brake actuation timing during the gait cycle is shown in Figure 1.

### 3.2. System Overview

**Figure 2:**
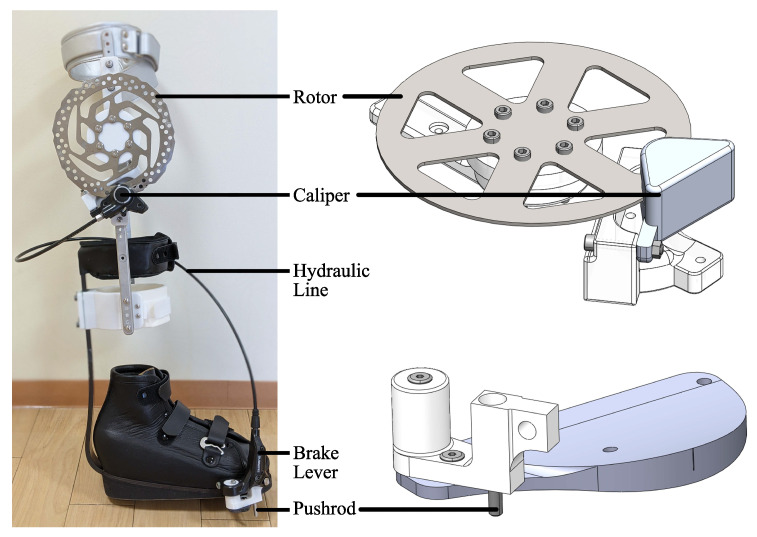
Exoskeleton device with the custom shoe attached and CAD models.

The frame of the exoskeleton is a commercial knee ankle foot orthosis (KAFO). From there, a hydraulic disc brake lever and caliper (Shimano BL-M365 and BR-M365, Shimano, Osaka, Japan) were modified and incorporated into the frame. The pushrod of the brake lever became the main actuating mechanism of the device and has a maximum travel distance of 5 mm. Three support structures were 3D printed on a Fortus 360mc (Stratasys Ltd., Rehovot, Israel) to mount the rotor, caliper, and brake actuator onto the KAFO. The parts were printed using a polycarbonate thermoplastic due to it’s high tensile strength (40 MPa XZ axis, 30 MPa ZX axis) and flexural strength (89 MPa XZ axis, 68 MPa ZX axis). The rotor and caliper were both mounted to the lateral rail of the KAFO. The brake actuator was attached to the sole of the shoe. Soft leather straps combined with hook-and-loop fasteners were used to ensure a comfortable and secure fit for the user when wearing the device. For the experiment that involved an individual with CP, a shoe fitted with a Carbon Ankle seven (Ottobock, Duderstadt, Germany) was used to replace the standard shoe of the KAFO. This shoe was custom molded for the individual’s foot. The key components of the brace are highlighted in Figure 2. Table 2 shows the weight of the different components. Excluding the custom shoe worn by the patient, the device itself weighs 1.25 kg. This is lighter than most of the previously mentioned exoskeletons. Additionally, excluding the shoe, all the parts used in constructing this device are either commercially available or 3D printed. This makes the device more economical and accessible.

### 3.3. System Modeling

**Figure 3:**
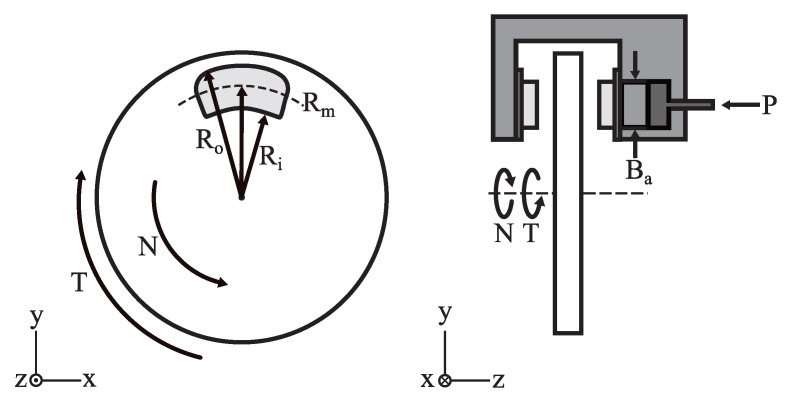
Forces acting on the rotor and caliper.

The torque produced by the hydraulic disc brake system can be modeled by (1) and (2). *T* is the brake torque generated (Nm), *P* is the applied brake pressure (Pa), *N* is the wheel speed (m/s), $N_{pads}$ is the number of brake pads in the assembly, μ is coefficient of static/kinetic friction between the disc pad and rotor, $B_{a}$ is the brake actuator bore diameter (m), $R_{m}$ is the mean radius of the brake pad (m), $R_{o}$ is the outer radius of the brake pad (m), and $R_{i}$ is the inner radius of the brake pad (m) [20]. Figure 3 shows a diagram of the forces acting on the rotor and caliper. Due to the low average walking speed and the brake being activated during the stance phase, *N* will be assumed to be 0 for the theoretical calculations and the coefficient of static friction, 0.4, will be used for the brake torque measurement. For the brake system used in this study, $N_{pads}$ is 2, $B_{a}$ is 0.022 m, $R_{o}$ is 0.079 m, and $R_{i}$ is 0.07 m.

(1) $$T=\left\{\begin{array}{cc} \frac{\mu_{k}P\pi B_{a}^{2}R_{m}N_{pads}}{4}, & \mathrm{when}\;N\neq \;0\\ \frac{\mu_{s}P\pi B_{a}^{2}R_{m}N_{pads}}{4}, & \mathrm{when}\;N=0 \end{array}\right.$$

(2) $$R_{m}=\frac{R_{o}+R_{i}}{2}$$

## 4. Performance Evaluations

### 4.1. Brake Torque Measurement

**Figure 4:**
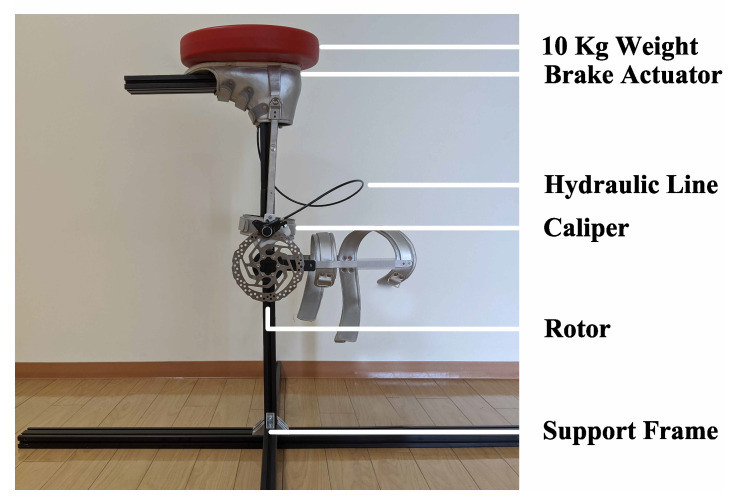
Experimental setup for measuring the maximum and dynamic braking torque. The exoskeleton is inverted in this setup.

Experiments were first performed to measure both the maximum braking torque and the dynamic braking torque produced by the device. The objective was to determine the relationship between the applied force due to the user’s body weight and the braking torque that would be used to support the knee joint. To measure these torques experimentally, a support structure was constructed using T-slot aluminum framing. The knee brace was then inverted and mounted on the structure, Figure 4. The device was mounted in this orientation so that forces mimicking the load of a human body could be easily applied to the brake actuator’s pushrod.

**Figure 5:**
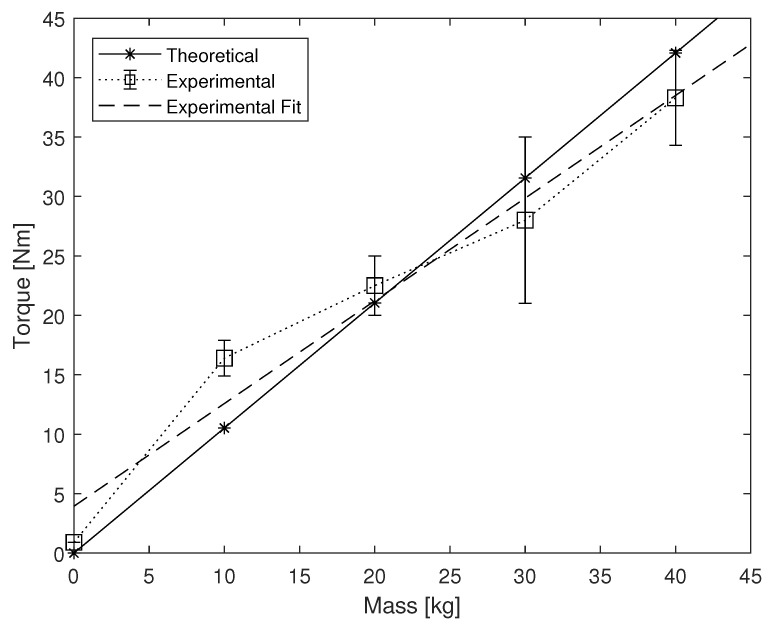
Maximum recorded braking torque before slipping occurred.

The first test was to measure the maximum braking torque, the maximum torque before slipping occurred. The experimental procedure is as follows: (i) the device was set in a vertical position, (ii) a weight ranging from 0 to 40 kg was placed on the pushrod, (iii) a force sensor (ZP-1000N, Imada, Toyohashi, Japan) attached to the brace 30 cm from the center of rotation was pulled perpendicularly until slipping occurred, (iv) the peak force measurement was recorded, and (v) the maximum braking torque was calculated. The results are shown in Figure 5. The device performed similarly to the theoretical calculations given by (1) and (2). At lower weights the experimental torque outperforms the theoretical torque. This may be due to the slight misalignment of the system components that creates extra friction and becomes negligible under higher loads. There should exist a maximum possible torque with the system, but this was not achieved with the current experimental setup.

The second test was to measure the dynamic braking torque. The procedure is as follows: (i) the device is positioned in a right-angle configuration, (ii) a weight ranging from 12 to 47 kg was added to the pushrod, (iii) a 5 kg weight was added 30 cm from the center of rotation on the KAFO, (iv) the weight attached to the KAFO was then released and allowed to free fall, (v) motion capture was used to measure the kinematics, and (vi) the dynamic braking torque was calculated based on the motion Equation (3).

(3) $$\tau_{F}=-ML^{2}\ddot{\theta }+MgL\sin\theta$$

**Figure 6:**
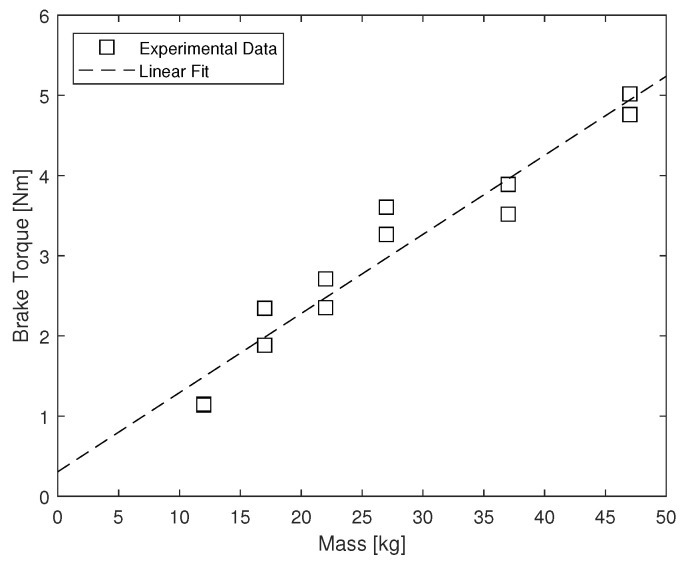
Dynamic braking torque.

$\tau_{F}$ is the braking torque due to friction (Nm), *M* is the mass attached to the device (kg), *L* is the length of the moment arm (m), θ is the angle between the vertical and the support (rad), and $\ddot{\theta }$ is the angular acceleration of the support (rad/s${}^{2}$). The results are shown in Figure 6. The dynamic brake torque increases linearly with the mass applied to the brake actuator.

It should be noted that these experiments show the best possible performance due to the fact that force was applied directly to the pushrod. The torques may vary from user to user due to the fact that we cannot verify how much force is contributed to the pushrod because people balance their weight differently when walking and standing.

### 4.2. Brake Activation Timing

**Figure 7:**
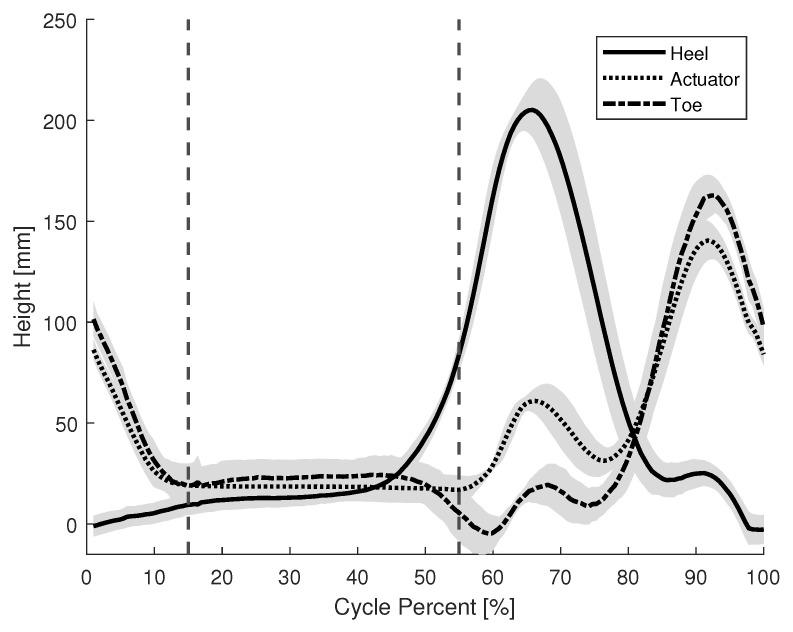
Brake activation timing during the gait of a healthy participant.

The purpose of this experiment was to verify the activation and deactivation timing of the brake mechanism during gait. An optical motion capture system (VICON MX, Vicon, Oxford, UK) was used to measure the kinematics. Reflective markers were placed on three key points on the device: the heel, actuator, and toe. The actuator was located near the metatarsals. The average height of these markers during a healthy participant’s gait with respect to the floor was measured. These points allow us to visualize the time in which the actuator made contact with the ground and, therefore, represent the period in which the brake is engaged. Figure 7 shows the average recorded height of each marker during the gait cycle. The vertical lines denote the period during the gait cycle where the brake mechanism is fully engaged; roughly 40% of the cycle.

## 5. Experimental Evaluations

### 5.1. Gait Support Experiment: Healthy Participants

The objective of this experiment was (i) to verify the basic mechanism of the device in applying braking torque to the knee joint, and (ii) to explore the relationship between the location of the push rod and the provided support.

Three healthy adult males volunteered for this experiment at the University of Tsukuba Hospital. The participants did not have any physical or neurological movement related disorders. All participants provided written consent for the experiment. The task in the experiment was to walk in a straight line at a normal pace for 10 m under different conditions. Five different conditions were designed in order to find the proper mounting location of the push rod. The conditions are as follows: (1) the push rod mounted to the lateral side of the insole, (2) the push rod mounted to the medial side, (3) the push rod mounted to the heel, (4) the device without the actuator, and (5) no device at all. The participants were asked to complete each task twice.

**Figure 8:**
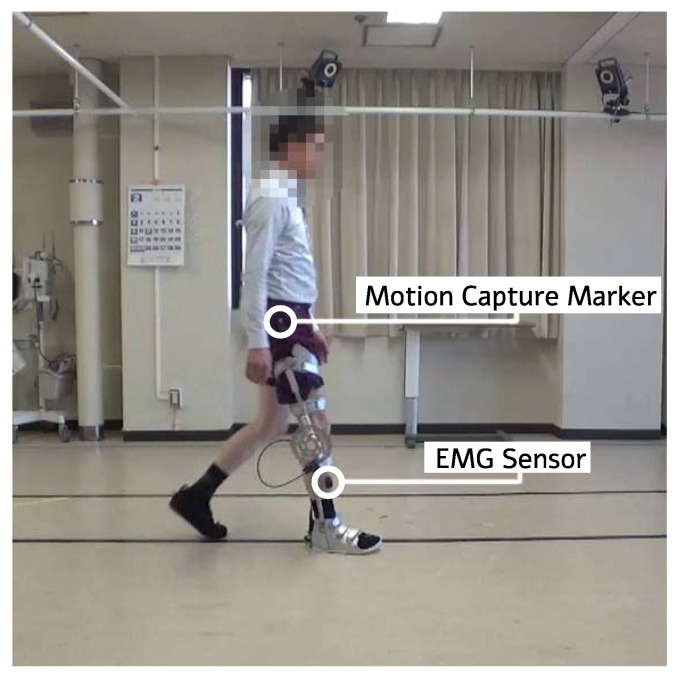
A healthy participant performing the experiment while wearing the device. One example of the motion capture marker and EMG sensor have been highlighted for each. Faces have been blurred for privacy.

In order to obtain the lower limb kinematics and muscle activation of the participants during the experiment, we used an optical motion capture system (VICON MX, Vicon, Oxford, UK) and a wireless electromyography (EMG) system (Trigno Lab, Delsys, Natick, MA, USA). Reflective markers were attached to the participants’ lower limbs to record the variation of the knee joint angle during gait with the motion capture system. The plug-in gait lower body model was used as the marker set. The muscle activation of the *vastus lateralis* (VL), *hamstring* (HAM), *tibialis anterior* (TA), and *gastrocnemius* (GAS) were recorded using four EMG sensors. These muscles were chosen because they contribute to the knee extension, knee flexion, ankle dorsiflexion, and ankle plantar flexion, respectively. During the experiment the EMG data is wirelessly transmitted to the system. This allows the participants to walk freely without their gait being encumbered by wires. The Trigno EMG sensors have on-board signal processing and help filter out preliminary noise. These data are synchronized with the motion capture data and then exported. The EMG data was then again filtered using a band-pass filter with low and high cut off frequencies of 30 and 400 Hz, rectified, and integrated using MATLAB code. The motion capture data was first cleaned up using Vicon’s Nexus 2.0 software (Vicon, Oxford, UK). During the motion capture process there are occasional optical occlusions caused by the user’s gait, clothing, or position in relation to the cameras. These occlusions can be interpolated from the preexisting geometry and kinematic data. After all optical occlusions have been removed and a continuous gait model achieved, the data of marker positions and sagittal lower limb joint angles are exported to a CSV file. These data are then imported into MATLAB. From there, the gait cycles were identified, and the duration of the gait cycles were normalized for all kinematic and filtered EMG data of the participants. The gait speed was measured using markers on the floor and a video camera. A picture of one of the participants performing the task while wearing the device is shown in Figure 8.

### 5.2. Gait Support Experiment: Individual with Cerebral Palsy

The objective of this experiment was to verify the effectiveness of the device when used by an individual with CP. For this pilot study, a 21-year-old male with CP (height: 160 cm, weight: 55 kg) volunteered to participate. He has paraplegia and his paralysis is right side dominant. The experiment was conducted at the University of Tsukuba Hospital under the careful supervision of a team of physicians. Informed consent was received, and the experiment’s procedures were approved by the University of Tsukuba Hospital institutional review board (H29-093).

**Figure 9:**
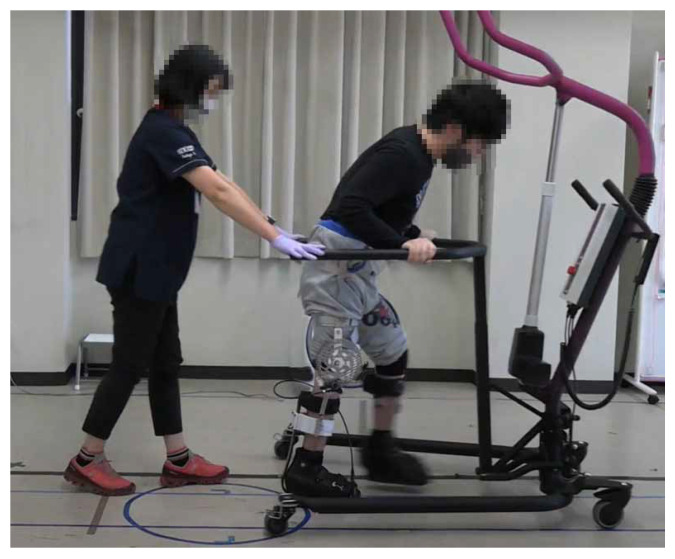
Individual with CP performing the experiment while wearing the device. Faces have been blurred for privacy.

The task during the experiment was to walk in a straight line for 6 m at a regular pace. The task was completed twice both with and without the device. The device was worn only on the participant’s right leg and the actuator was placed on the lateral side of the foot by the metatarsals. The healthy participant study showed that the pushrod’s location and provided support are related to the user’s gait. Therefore, this location was chosen in an attempt to reduce interference and maximize the force transfer to the pushrod based on the typical crouch gait walking pattern [21]. The participant wore the same carbon fiber shoe that was used in the construction of the device on his left leg for balance. The participant uses an electric wheelchair in his daily life and required extra support during the experiment. His upper body was supported by a walker during the experiments. A medical doctor was also present to guide the participant and keep them moving in a straight line. A picture of the individual during the experiment can be seen in Figure 9.

The motion capture and EMG system were used again to obtain the lower limb kinematics and muscle activation of the participant. The muscle activation of the *tensor fascia latae* (TFL), *hamstring* (HAM), *gastrocnemius* (GAS), *tibialis anterior* (TA), *quadriceps* (QUAD), and *gluteus maximus* (Gmax) were measured on the left and right sides of the body. The EMG data was filtered, rectified, and integrated. Gait cycles were identified using the motion capture system and the duration of the gait cycle was normalized.

## 6. Results

### 6.1. Gait Support Experiment: Healthy Participants

**Figure 10:**
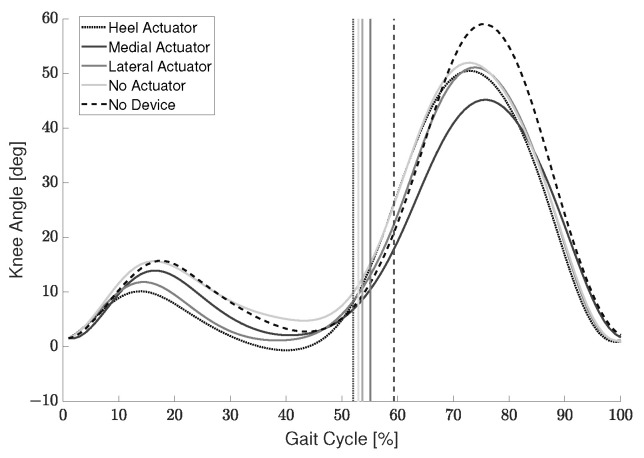
Average healthy participant gait cycle for each condition. Vertical lines represent the end of the stance phase.

Persons with crouch gait experience difficulty while walking due to excessive knee flexion. Therefore, one metric used to measure the support provided by the device was the reduction in knee angle during stance. Looking at Table 3, the average reduction in knee joint angle compared to the condition without using the device is shown for each participant. The largest reduction for each participant is shown in bold. Two of the three participants experienced the largest knee joint angle reduction when the actuator was located on the heel. This result makes sense as most healthy participants place most of their body weight on their heel during the initial contact and mid-stance gait phases. Figure 10 shows the average gait cycle of the healthy participants’ right leg for each condition. There is a slight reduction in knee angle during swing and the swing phase occurs earlier when the device is used. This occurs for all conditions with the device even when the actuator is not engaged. An unpaired *t*-test was performed using the maximum and minimum knee flexion angle during the stance phase of the three participants. Each condition was compared to the no device condition. The heel actuator reduced the flexion angle by a maximum of 9.0${}^{\circ }$ (*p* = 0.0052), the medial actuator by 7.6${}^{\circ }$ (*p* = 0.013), and the lateral actuator by 9.2${}^{\circ }$ (*p* = 0.0044), while the no actuator condition did not (*p* = 0.18). This shows that using the actuator the flexion angle is reduced with *p* < 0.05, but not when using only the KAFO frame without the actuator engaged. The participants were instructed to walk at a normal pace. The average walking speed for all participants while wearing the device was 1.36 ± 0.20 m/s and 1.50 ± 0.29 m/s while not wearing the device.

In order to evaluate the performance of the device in terms of muscle activation reduction during gait, an index termed the Reduction Ratio (RR) was calculated, (4).

(4) $$\mathrm{RR}=\frac{EMG_{NoDevice}-EMG_{Device}}{EMG_{NoDevice}}$$

**Figure 11:**
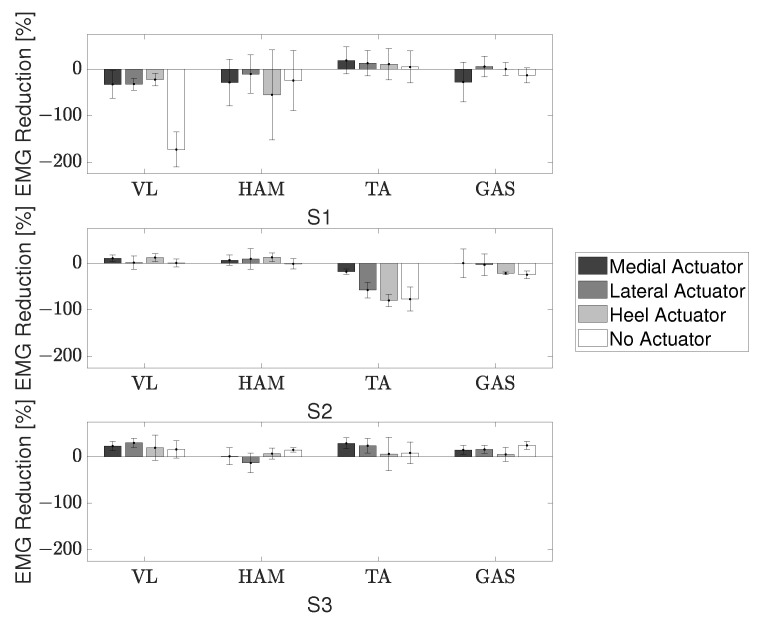
EMG reduction ratio during stance for healthy participants.

RR was calculated using the device with a medial actuator, lateral actuator, heel actuator, and no actuator. The terms $EMG_{Device}$ and $EMG_{NoDevice}$ were calculated by integrating the area under the averaged EMG curve. Two of the three participants showed positive values of RR, a reduction in muscle activation, for the VL and HAM muscles, Figure 11.

### 6.2. Gait Support Experiment: Individual with Cerebral Palsy

**Figure 12:**
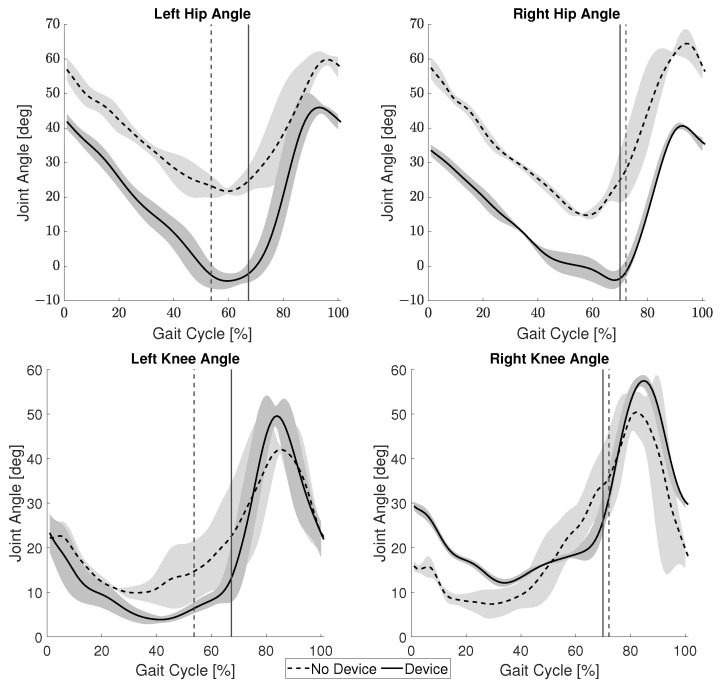
Joint angles of the individual with CP. Vertical lines represent the end of the stance phase.

The average angles of the hip and knee joints during the gait cycle for both conditions are presented in Figure 12. The vertical line indicates the toe-off point in the gait cycle. The toe-off of the left leg occurred roughly 14% later in the gait cycle when the participant wore the device. However, there was no significant change in the toe-off timing of the right leg. There was an average reduction in hip joint angle of 18.8 ± 5.2${}^{\circ }$ and 21.7 ± 4.2${}^{\circ }$ for the left and right sides, respectively. The left knee experienced a smaller average angle when using the device, but the right knee showed a smaller average angle when not using the device. Inversely, the average variation, difference between the minimum and maximum recorded angles, during the stance phase was less for the left knee without the device and less for the right knee with the device, while a lower minimum angle is important for reducing crouch gait, the variation during stance can be an indicator of the user’s overall stability.

**Figure 13:**
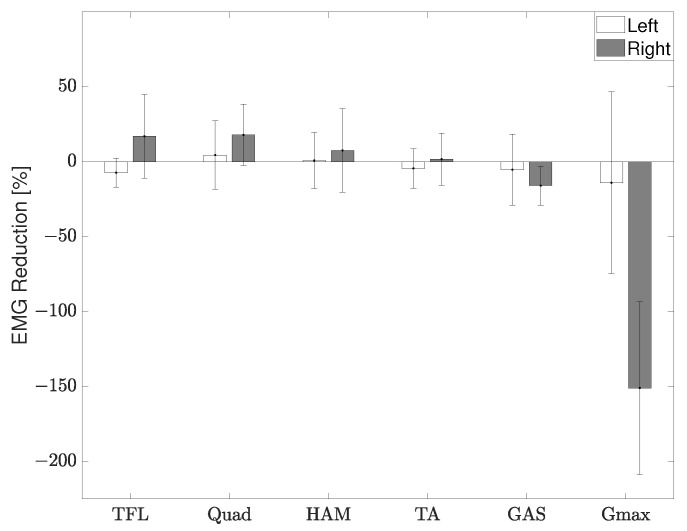
EMG reduction ratio of the measured muscle groups during stance for an individual with CP.

The RR was again calculated by integrating the EMG during stance for the two conditions using (4), Figure 13. There was a reduction in muscle activation of the right leg, the leg the device was attached to, for the TFL, Quad, HAM, and TA muscles. However, a large increase in muscle activation was noticed for the Gmax muscle on the right leg. The co-activation index was then calculated and showed a reduction from 0.48 to 0.24 on the right side and from 0.17 to 0.017 on the left side. This calculation did not include the initial contact because it is normal to have a high co-activation to deal with the ground impact even in a healthy gait.

**Figure 14:**
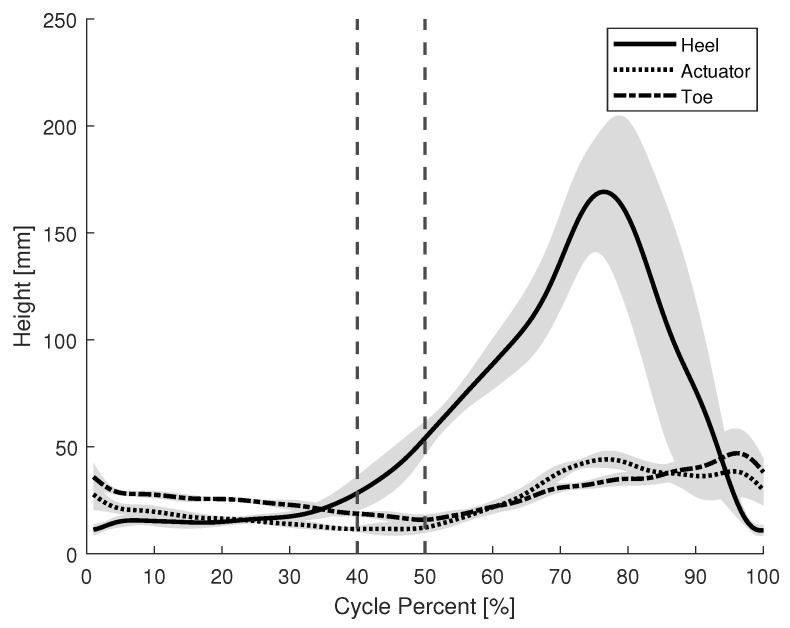
Brake activation timing during the gait of an individual with CP.

Lastly, the timing of the brake activation was also recorded for the gait of the individual with CP. Figure 14 shows the average height of the heel, toe, and actuator from the floor during the gait cycle. The vertical lines denote the period that the brake mechanism is fully engaged; about 10% of the gait cycle. There was no impairment to propulsion as the participant walked at an average pace of 0.36 m/s with the device and 0.37 m/s without the device. The average step length was 0.33 m both with and without the device.

## 7. Discussion

### 7.1. Device Evaluation: Healthy Participants

Looking at Figure 11, participant S1’s data showed a decrease in performance, corresponding to an increase in VL and HAM muscle activation, when using the device compared to when walking without it. During the trials, S1 appeared to actively resist the braking efforts of the device. This could have been a possible reason for the increased muscle activation when using the brake actuator. Additionally, for the trial that involved using the device without the actuator, S1 showed a large decrease in performance. This phenomenon is attributed to the order in which the conditions were tested. The participants performed the actuated trials first. After the actuator was removed, S1 walked with an exaggerated motion as if to test their full range of motion due to now being free of the braking constraints. This idea is also supported by the fact that S1 showed a large increase in knee joint angle during the no actuator condition. Though the sample size was small, the data was sufficient to verify that (i) the mechanism is capable of applying a braking torque to the knee joint, and (ii) the location and support provided by the pushrod are related to the user’s gait.

### 7.2. Device Evaluation: Individual with Cerebral Palsy

Figure 12 shows a decrease in hip angle for both the left and right side. Since one symptom of crouch gait is excessive flexion of the hip joint, this suggests the participant’s crouch gait was partially ameliorated when wearing the device. The reduction in hip angle of both sides was interesting due to the fact that the device was only worn on the right leg. Since the participant’s paralysis was right leg dominant, it appears the device was able to better support his gait.

The right Gmax in Figure 13 shows that the participant actually exerts more energy during the stance phase when wearing the device as opposed to the trial without it. However, this may not necessarily be a negative aspect of the device. During stance, the Gmax muscle can be used to support oneself and the swing phase of the opposite leg. It is possible that by using this device, the participant is now able to better use their muscles to support themselves during gait. This is reinforced by the improved swing motion of their left leg, Figure 12. The increased muscle activation of the Gmax may also be a reason for the reduction in hip flexion. Additionally, paralysis of ankle joint control is widely observed in persons with CP and the ground reaction force during stance is significantly lower in CP gait than healthy gait [22]. In combination with the rigid KAFO worn by the participant, the change in the Gmax muscle suggests the device provides support instead of a hindrance. Further studies with a larger group of individuals with CP will be needed to verify these results. A reduction in the co-activation index was shown for both the right and left side. During stance phase when the activation of the extensor muscles is needed, involuntary activation of the flexor muscles also occurs in individuals with CP. Therefore, this reduction in co-activation further suggests an improvement of gait.

Comparing the activation timing of a healthy participant, Figure 7, and an individual with CP, Figure 14, the brake is fully engaged for about 40% and 10% of the gait cycle, respectfully. An actuator that makes use of the entire surface area of the shoe, instead of a single point, may be preferable to provide a longer period of brake actuation.

As this experiment involved a case study with an individual with CP, it is important to also take their feedback into consideration along with the quantitative data. They commented that they felt the braking force produced by the knee brace and began to trust it to support themselves while walking. This relates back to one of our initial concepts for the device. We wanted the user and exoskeleton to form a cooperative control strategy. The passive gait-based sensing appears to lend itself better to a more natural and synchronous operation of the device. Even if a device is mechanically sound, the individual needs confidence in the device for it to be truly beneficial. This could be an important concept in not just exoskeletons, but other health care applications as well. Being labeled as a patient can bring to mind many feelings such as helplessness and lack of control. Allowing the individual to feel more involved and in control of what happens with their body, may lead to better health care practices overall.

### 7.3. Gait Support

While the device was able to mitigate the knee flexion during stance, it was not able to lock the knee joint completely. This complete locking may be necessary for certain individuals with CP. These cases would be for patients who exhibit more than 40 degrees of knee flexion during their stance [9]. For less severe cases, this device has the potential to benefit their gait. The nature of the mechanism’s design allows it to only be actuated when the foot remains on the ground during the stance phase. The quick activation and deactivation time of the brake, relative to a person’s average walking speed, allows for an effective control solution that does not inhibit the user. Exoskeletons that rely on sensors for timing the actuation of a braking mechanism have the potential for errors [12,23]. A robust design, such as the presented passive mechanism, provides a safer platform for the user. These experiments were conducted in a controlled environment on flat, even ground. Since the mechanism is most effective when the pushrod is fully depressed, it is expected that there would be a reduction in provided support if used on surfaces that are soft or slanted.

### 7.4. Comparison to Related Work

The first objective of this study was to investigate the effects of adding a braking force to the knee joint during stance. The device reduced the hip and knee flexion for most participants and was able to benefit specific muscle groups. Compared to the commercially produced exoskeletons, our device was not able to outperform them. The HAL is designed specifically for rehabilitation and can offer full support for a user’s body weight as they progressively regain their abilities [16]. Our device could not fully support the participant with CP alone. They required the additional support of a walker when performing the experiment. However, our device showed comparable performance to the active exoskeleton created by Yamada et al. in terms of support and energy usage [13]. Our device also outperformed the passive exoskeleton of Van Dijk et al., as their study did not show any significant reduction in energy usage [18].

The second objective of this work was to propose a compact, lightweight, and simple mechanism for supporting exoskeleton joints. The most expensive components in this device were the hydraulic disc brake and KAFO. However, these components are both commercial and widely accessible making it an appropriate technology for many parts of the world. Compared to the two commercial exoskeletons presented in this paper, it is a significantly more cost effective option. The total weight of the our passive exoskeleton is 2.2 kg with the carbon fiber shoe worn by the participant with CP and 1.25 kg without it. Without the carbon fiber shoe, the proposed device is the lightest of all the previously compared exoskeleton robots. With the addition of this custom shoe, the device is ranked in the middle with respect to weight. The simple mechanism also allows the device to be easily adjusted to accommodate different users.

The last objective was verifying the gait-based control system for an individual with CP. Our control system was not able to match the commercial products. The HAL uses EMG sensors to measure muscle activity and infer the intention of the user. This is not a capability our passive system would be able to achieve. Most of the related research showed accurate sensing and actuation capabilities. Our system managed to actuate every time without error. The gait-based control system was sufficient in providing support for the participants in this study.

### 7.5. Future Improvements

Optimizations to the system overall are also currently being considered. The polycarbonate mounts held up well from repeated use during the experiments and no visible faults were found. The mounts were over designed for safety, but it is possible to modify the design to reduce the weight of the components. The rotor that the knee brake uses is an unmodified bicycle rotor, while effective, it will be better to shrink the size of the rotor for practical reasons. Currently, our device is only designed for the right leg. We would also like to build a mirrored system for the left leg and perform more experiments with participants wearing both devices simultaneously.

Future improvements will be made to refine the design in an attempt to maximize the stance support provided by the passive knee exoskeleton. The current pushrod for the brake system only acts at a single point on the foot. Increasing the area of actuation could allow for a longer and more consistent braking force to be applied. One possible method of achieving this would be to replace the current actuator with a fluid filled insole that connects to the hydraulic line. Additionally, unlike an active exoskeleton, the support mechanism is not easily disengaged. If the user is in stance, the brake is engaged and would inhibit the user from squatting down. As this is a convenient action in daily life, it would be beneficial to design a component that could temporarily disable this mechanism.

While this research was targeted towards persons with CP, it is possible that other individuals with similar walking disabilities could benefit from this device as well. For instance, there are often drastic changes in gait for persons that are recovering from stroke [24]. If such a patient were experiencing difficulties during stance, a passive gait-based solution could benefit their gait.

## 8. Conclusions

Using commercial and 3D printed components, we created a cost effective and lightweight exoskeleton that has shown potential to improve crouch gait by adding a braking force to the knee joint during stance. The device was able to reduce the hip and knee flexion for most participants and had benefits for specific muscle groups. The gait-based control needs to be improved for use by individuals with CP to prolong the period in which the brake is fully engaged.

## Figures and Tables

**Table 1:** Exoskeleton summary.

Exoskeleton	Actuator	Support Mechanism	Power	Weight (kg)
Lerner [12]	Active	DC Motor	Battery/External	3.2
Yamada [13]	Active	DC Motor	Battery	1.76
Shideler [14]	Semi-Active	NMES	Battery	3.2
C-Brace * [15]	Active	Hydraulic	Battery	1.39
HAL * [16]	Active	DC Motor	Battery	14
Wang [17]	Passive	Spring	-	1.95
Van Dijk [18]	Passive	Spring	-	12

* Commercially available exoskeletons.

**Table 2:** Weight of Assembly Components.

Component	Weight (kg)
Commercial KAFO	0.50
Carbon Fiber Shoe	0.95
Caliper/Actuator	0.25
Rotor	0.14
Polycarbonate Mounts	0.28
Total *	2.2

* Total includes the weight of additional fasteners.

**Table 3:** Average difference in knee angle vs. actuator location during stance.

	S1	S2	S3
Medial	0.13${}^{\circ }$	2.11${}^{\circ }$	2.64${}^{\circ }$
Lateral	0.51${}^{\circ }$	5.67${}^{\circ }$	**5.43${}^{\circ }$**
Heel	**2.93${}^{\circ }$**	**8.95${}^{\circ }$**	4.82${}^{\circ }$
No Actuator	−10.21${}^{\circ }$	2.89${}^{\circ }$	5.04${}^{\circ }$

The largest reduction for each participant is shown in bold.

## Data Availability

The data presented in this study is available on reasonable request from the corresponding author. The data is not publicly available due to patient privacy.

## Abbreviations

The following abbreviations are used in this manuscript:

CP	cerebral palsy
CSV	comma-separated values
EMG	electromyography
GAS	gastrocnemius
Gmax	gluteus maximus
HAM	hamstring
KAFO	knee ankle foot orthosis
NMES	neuromuscular electrical stimulation
QUAD	quadriceps
RR	reduction ratio
TA	tibialis anterior
TFL	tensor fascia latae
VL	vastus lateralis
